# Expression of programmed cell death ligand 1 (PD-L1) and prevalence of tumor-infiltrating lymphocytes (TILs) in chordoma

**DOI:** 10.18632/oncotarget.3576

**Published:** 2015-03-14

**Authors:** Yong Feng, Jacson Shen, Yan Gao, Yunfei Liao, Gregory Cote, Edwin Choy, Ivan Chebib, Henry Mankin, Francis Hornicek, Zhenfeng Duan

**Affiliations:** ^1^ Sarcoma Biology Laboratory, Department of Orthopaedic Surgery, Massachusetts General Hospital and Harvard Medical School, Jackson, Boston, Massachusetts, USA; ^2^ Department of Orthopaedic Surgery, Union Hospital, Tongji Medical College, Huazhong University of Science and Technology, Wuhan, China; ^3^ Department of Endocrine, Union Hospital, Tongji Medical College, Huazhong University of Science and Technology, Wuhan, China; ^4^ Division of Hematology and Oncology, Massachusetts General Hospital and Harvard Medical School, Boston, Massachusetts, USA; ^5^ Department of Pathology, Massachusetts General Hospital and Harvard Medical School, Boston, Massachusetts, USA

**Keywords:** PD-L1, TILs, chordoma, immunotherapy

## Abstract

Chordomas are primary malignant tumors of the notochord that are resistant to conventional chemotherapy. Expression of programmed cell death ligand 1 (PD-L1), prevalence of tumor-infiltrating lymphocytes (TILs), and their clinical relevance in chordoma remain unknown. We evaluated PD-L1 expression in three chordoma cell lines and nine chordoma tissue samples by western blot. Immunohistochemical staining was performed on a chordoma tissue microarray (TMA) that contained 78 tissue specimens. We also correlated the expression of PD-L1 and TILs with clinical outcomes. PD-L1 protein expression was demonstrated to be induced by IFN-γ in both UCH1 and UCH2 cell lines. Across nine human chordoma tissue samples, PD-L1 protein was differentially expressed. 94.9% of chordoma samples showed positive PD-L1 expression in the TMA. The expression score of PD-L1 for metastatic chordoma tumors was significant higher as compared with non-metastatic chordoma tumors. Expression of PD-L1 protein significantly correlates with the presence of elevated TILs, which correlates with metastasis. In summary, our study showed high levels of PD-L1 are expressed in chordoma, which is correlated with the prevalence of TILs. The current study suggests targeting PD-L1 may be a novel immunotherapeutic strategy for chordoma clinical trials.

## INTRODUCTION

Chordoma, an extremely rare cancer presumably originating from notochord, accounts for 20% of primary spine tumors and 1- 4% of primary malignant bone tumors [1, 2]. Epidemiological studies suggest that chordoma affects roughly one in a million individuals; approximately 300 new cases of chordoma are diagnosed in the US per year with an overall median survival of about six years [3]. The standard treatment for these tumors is en-bloc resection accompanied by radiotherapy given both before and after surgery; however, the critical anatomic location (spread along critical bony and neural structures) and the commonly large tumor size make clinical management of these patients difficult. Unlike osteosarcoma, which is relatively sensitive to chemotherapeutic agents, chordoma is resistant to chemotherapy. Distant metastasis occurs in 20-40% of patients with chordoma of the spine and in less than 10% of patients with skull-base tumors, with the median survival time after metastasis around one year [3-5]. Therefore, development of novel therapeutic strategies is critical for this patient population.

There is growing interest in the immunoregulatory receptor programmed cell death 1 (PD-1) and the corresponding B7 family of ligands as a pivotal mechanism of tumor immune tolerance and escape in cancer. PD-1 is a member of the B7-CD28 family that co-regulates T-cell receptors and contributes inhibitory signals to mediate physiological immune escape and tolerance [6, 7]. PD-1 is expressed in various immune cell types and its activation attenuates T-cell function, survival, and expansion [8, 9]. Programmed death-ligand 1 (PD-L1) is a transmembrane protein that has been shown to be expressed in different tumor cells, and play an important role in suppressing the immune function in diseases like cancer. Expression of PD-L1 by tumor cells is believed to mediate the inhibition of local immune responses, thus shielding the tumor from T-cell mediated killing.

Tumor-infiltrating lymphocytes (TIL) in many cancers express PD-1 and have been shown to be correlated with high PD-L1 expression in different cancers [10-15]. In the inflammatory microenvironment, stimuli such as IFN-γ may up regulate PD-L1 expression in peripheral tissues and immune cells to repress the immune response [16-18]. Interestingly, tumors from diverse locations can co-opt this checkpoint system by up regulating PD-L1 expression constitutively or in response to inflammation, including breast, ovarian, gastric, pancreatic, lung, and renal cell carcinomas [19-23]. However, PD-L1 expression and prevalence of tumor TILs in chordoma are unknown.

The aim of our study is to investigate the expression level of PD-L1, TILs, and clinicopathological parameters in chordoma, with a particular focus on any potential correlation with prognosis.

## RESULTS

### Expression of PD-L1 in chordoma cell lines

**Figure 1:**
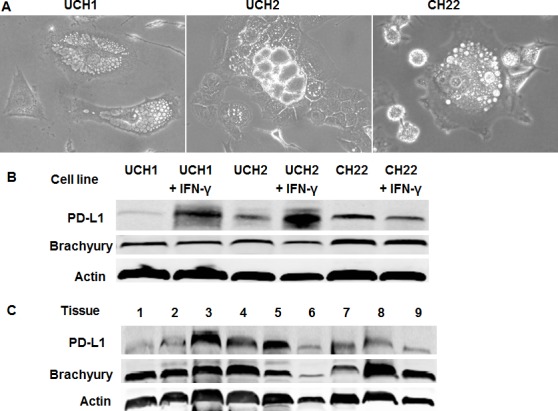
PD-L1 expression in chordoma (A) Cell morphology and growth characteristics of chordoma cell lines. UCH1, UCH2, and CH22 cells exhibited round nuclei with clear vacuolated cytoplasm. (B) PD-L1 expression in chordoma cell lines. PD-L1 protein expressions were induced 16-fold and 4-fold by IFN-γ in UCH1 and UCH2 cell lines, respectively. (C) PD-L1 expression in chordoma tissues. Relative expressions of PD-L1 were present in 9 chordoma specimens. PD-L1 expression was evaluated from total protein by western blot and absolute expression of PD-L1 was normalized to β-actin. Three of nine samples were found to have high expression.

Morphology of chordoma cells usually consist of small non-vacuolated cells, intermediate cells with a wide range of vacuolization, and large heavily vacuolated (physaliferous) cells [24, 25]. UCH1, UCH2, and CH22 cells exhibited characteristics of round nuclei with clear vacuolated cytoplasm (Figure 1A). The expressions of PD-L1 were analyzed by western blot in chordoma cell lines. PD-L1 was constitutively expressed in UCH1, UCH2, and CH22 cells (Figure 1B). PD-L1 protein expressions were induced 16-fold and 4-fold by IFN-γ in UCH1 and UCH2 cell lines, respectively, while there was no significant response to IFN-γ in the CH22 cell line. Brachyury protein expression remained at nearly the same level compared with the β-actin expression in all chordoma cell lines.

### PD-L1 expression in chordoma tumor tissue

To validate the chordoma cell line data, the PD-L1 western blot assay was performed on total protein isolated from 9 chordoma human tumor samples (Figure 1C). Brachyury protein expression was present in all tumor samples. PD-L1 protein was also expressed in these samples. Absolute expression of PD-L1 was normalized to β-actin. Three of nine samples exhibited high expression. There were two samples with intermediate expression, and four samples with low expression.

### Correlation between PD-L1 expression and clinicopathology

**Figure 2:**
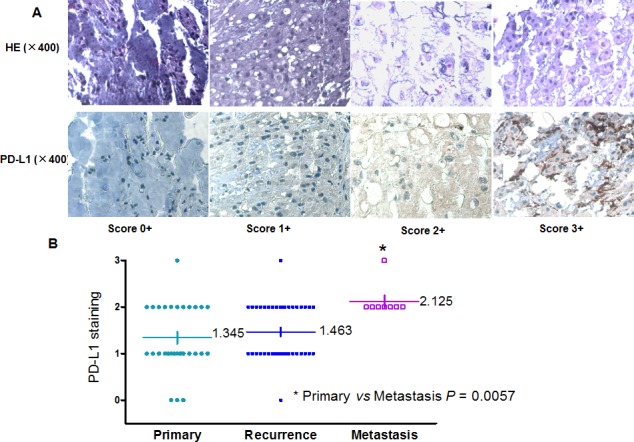
Association of PD-L1 expression with clinical outcome in chordoma (A) Representative images of different IHC staining intensities of PD-L1 and HE staining were shown in the chordoma TMA. (B) Distribution of PD-L1 staining scores among primary, recurrent, and metastatic tumor tissues. The median PD-L1 expression score for metastatic status (2.215) was significantly higher compared with primary status (*P* = 0.0057).

PD-L1 immunoreactivity was found in the cytoplasm of tumor cells. PD-L1 staining was detected in 78 TMA samples (including recurrence or metastasis) from 56 patients. The median age was 62 years (range: 24 - 85), and predominantly male (69.6% of patients). Of the 78 chordoma samples, 94.9% were positive for PD-L1 expression. Based on PD-L1 staining intensities in tumor samples, no staining (0) and weak staining (1+) specimens were classified as PD-L1-low patients (57.1%); moderate staining (2+) and intense staining (3+) specimens were classified as PD-L1-high patients (42.9%) (Figure 2A). We evaluated the clinicopathologic features of the human tumor samples and found no significant relationship between PD-L1 expression and age at surgery, gender, or tumor location. There was no statistical significance between the median PD-L1 expression score for the relapsed status (1.463) compared with the score for primary tumor (1.345); however, the median PD-L1 expression score for the metastatic group (2.215) was significantly higher compared with the primary group (*P* = 0.0057; Figure 2B).

### Prognostic value of PD-L1 expression

**Figure 3:**
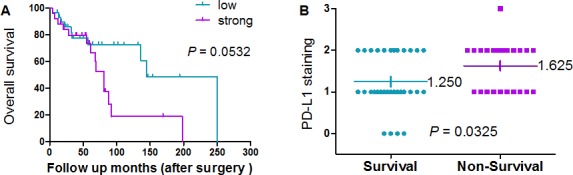
Prognostic value of PD-L1 expression in chordoma (A) Association between expression of PD-L1 (PD-L1 staining <2+ and PD-L1 staining >2+) and survival in chordoma patients. Kaplan–Meier survival analysis was used to analyze the correlation between the staining of PD-L1 expression and survival. The median overall survival for PD-L1-low patients compared with PD-L1-high patients exhibited a borderline trend, but did not achieve statistical significance. (B) Distribution of PD-L1 expression in survival and non-survival chordoma patient samples as determined by immunohistochemistry. The average PD-L1 expression levels for survivors and non-survivors were 1.250+ and 1.625+, respectively (*P* = 0.0325).

The median overall survival for patients with low PD-L1 expressing tumors was 145 months compared with 81 months for patients with high PD-L1 expressing tumors, exhibiting a borderline trend, but not achieving statistical significance (*P* = 0.0532; Figure 3A). We further analyzed the correlation between expression of PD-L1 and the prognosis of chordoma patients. The average expression levels of PD-L1 for survivors and non-survivors were 1.250+ to 1.625+, respectively (Figure 3B), and a statistically significant difference in expression PD-L1 expression was identified between these groups of patients (*P* = 0.0325).

### Associations between expression of PD-L1 and TILs

**Figure 4:**
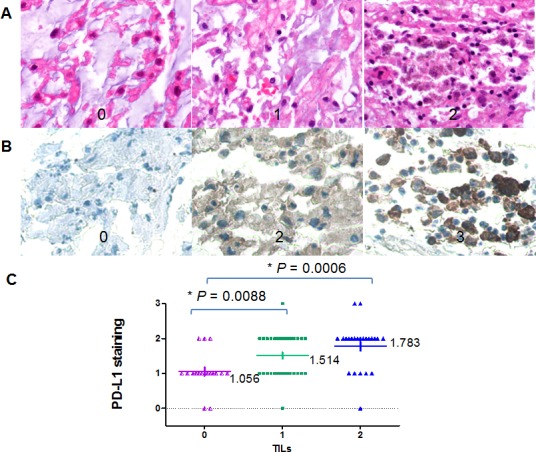
Correlation between PD-L1 expression and TILs by IHC (A) representative TILs in chordoma tissues (400×); score 0, no TILs; 1, rare/few TILs; 2, brisk/prominent TILs. (B) representative PD-L1expression by IHC in chordoma tissues (400×); (C) A significant positive correlation was shown between PD-L1 protein expression and TILs in patients with chordoma (*P* = 0.0088; *P =* 0.0006).

Representative pictures of chordoma cases showing different levels of TILs (scores 0 - 2+) were depicted in Figure 4A. Accordingly, representative pictures of PD-L1 expression were shown in Figure 4B. Based on the scoring criteria of TILs in tumor samples, no TILs were found in 23.1% cases (18/78). The number of cases with rare/few lymphocytic infiltrates (scores 1+) was 37 out of 78 samples (47.4%). The percentage of cases with brisk/prominent TILs was 29.5% (23/78). PD-L1 protein expression was significantly associated with the presence of elevated TILs. Cases with elevated TILs showed significantly higher expression levels of PD-L1 (Figure 4C, *P* = 0.0088; *P =* 0.0006).

### Relationships between TILs and clinicopathological features of chordoma

**Figure 5:**
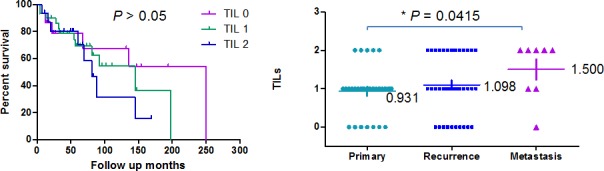
Relationships between clinicopathological features and TILs (A) Association between expression of TILs (score 0, no TILs; 1, rare/few TILs; 2, brisk/prominent TILs) and survival in chordoma patients. Kaplan–Meier survival analysis was used to analyze the correlation between the staining of TIL expression and survival. The median overall survival for TILs-low patients compared with TILs-high patients exhibited a borderline trend, but did not achieve statistical significance. (B) Distribution of TIL expression in survival and non-survival chordoma patient samples as determined by immunohistochemistry. The average TILs expression levels for the primary group and the metastasis group were 0.931+ and 1.500+, respectively (*P* = 0.0415).

We evaluated the clinicopathologic features of chordoma tumor samples and found no significant relationship between PD-L1 expression and age at surgery, gender, or tumor location. The presence of increased TILs was not significantly associated with survival. The median overall survival for TILs-few patients compared with TILs-prominent patients exhibited a borderline trend, but did not achieve statistical significance (Figure 5A). There was no statistical significance between the median PD-L1 expression score for recurrence status (1.089) compared with the score for primary status (0.931); however, the average TIL expression levels for the primary group and metastasis group were 0.931+ to 1.500+, respectively (*P* = 0.0415, Figure 5B).

## DISCUSSION

PD-L1 has been shown to be expressed in different solid tumors [19-23]. Our previous study showed that high levels of PD-L1 mRNA are expressed in a subset of osteosarcoma [26]. Many cancer cells contain PD-L1 mRNA but do not express PD-L1 protein [27]. We demonstrate that all chordoma cell lines UCH1, UCH2, and CH22 express PD-L1 protein. Consistent with previous studies that showed the ability of IFN-γ to up regulate expression of PD-L1 [28], treatment of UCH1 and UCH2 cell lines with IFN-γ induced significantly higher levels of PD-L1 protein expression. We further confirmed that PD-L1 is expressed at different levels in all 9 chordoma tumor tissue samples. Three of nine samples were found to have a high expression level of PD-L1. These varying expression levels of PD-L1 may result from different chordoma microenvironments.

Loss of tumor suppressor phosphatase and tensin homolog (PTEN) had been shown to increase PD-L1 expression and immunoresistance in glioma [29]. In primary glioma tissues, levels of PD-L1 protein correlated with PTEN loss, and tumor-specific T-cells lysed human glioma targets expressing wild-type PTEN more effectively than those expressing mutant PTEN, suggesting that loss of PTEN is associated with high levels of PD-L1 and immune evasion and immunoresistance [29-31]. Recently, another study also demonstrated that loss of Lkb1 and PTEN resulted in elevated PD-L1 expression in lung squamous cell carcinoma [32]. Our previous study had found that deficiency of PTEN expression (around 80% of chordoma tissues) represents a key aspect of chordoma pathogenesis [33]. Interestingly, two of the three chordoma lines (UCH1 and CH22) used in this study that showed high levels of PD-L1 expression also hold genetic changes of PTEN loss [25, 34]. Furthermore, some of these cell lines also harbor other genetic changes, such as loss of p16, SMARCB1, MTAP, or Lkb1, that make them potentially useful models for evaluating PD-L1 targeted immunotherapies [29-31, 35]. These studies suggest PTEN loss may be part of the potential mechanism of increased PD-L1 expression in chordoma. The most direct evidence for this hypothesis has been confirmed in a recent study [36], in which PTEN short hairpin RNA (shRNA) knockdown led to significantly higher cell-surface PD-L1 expression and PD-L1 transcripts, suggesting its involvement in transcriptional regulation.

In order to characterize the relationship between the expression level of PD-L1 protein and clinical behavior, we used a chordoma TMA that allowed the simultaneous characterization of the expression status of PD-L1 in 78 chordoma samples. Our study showed that 42.9% samples were classified as staining highly for PD-L1. In our analyses, there was no correlation of PD-L1 expression with age at surgery, gender, or tumor location. Our study showed the median PD-L1 expression score for the group with metastsis was significantly higher compared with the PD-L1 expression score for the group without metastasis. Furthermore, previous studies have demonstrated a strong correlation between the expression of PD-L1 and poor prognosis in different solid tumors, including glioblastoma, pancreas, ovarian, breast, renal cell carcinomas, head and neck squamous cell carcinomas, esophageal, and non-small cell lung cancers [27, 29, 37-41]. The longest length of follow up of chordoma patients in our current study was over 20 years. Kaplan–Meier survival analysis showed that there was a trend for poorer overall survival for chordoma patients with high expression of PD-L1. The average expression level of PD-L1 for non-survival patients was significantly higher than the survival patients group.

TILs induce expression of PD-L1 through up regulation of cytokines, such as IFN-γ [42]. In accordance with IFN-γ up regulating PD-L1 protein in chordoma cell lines, our chordoma TMA samples with increasing TILs also showed significantly higher PD-L1 expression. This finding was consistent with a recently published study on PD-L1 protein co-localizing with TILs [28]. We further analyzed the association between TILs and clinicopathological features. However, no significant difference between the median overall survival for TILs-zero patients with TILs-prominent or TILs-few patients was found. The average TIL expression level for the metastasis group was significantly higher than the primary group. Consistent with these findings, TIL infiltration could facilitate metastases in melanoma [14].

Multiple agents targeting the PD-1/PD-L1 system in cancer are currently at different stages of clinical development [43-46]. These promising clinical trials show that blockade of the PD-1/PD-L1 axis using monoclonal antibodies can reactivate the antitumor immune response and induce lasting clinical benefit in nearly one third of (heavily pretreated) patients with advanced melanoma, lung, and renal cell carcinomas [44, 46]. In these previously heavily treated patients, the response rates ranged from 18% to 28%, depending on the tumor histology, and importantly the responses were durable. Notably, tumor PD-L1 positivity scores determined by immunohistochemistry (IHC) were found to be predictive of response to the anti-PD-compound [44]. Most importantly, anti-PD-L1 treatment and irradiation synergistically promote anti-tumor immunity in mice [47], and acquired resistance to fractionated radiotherapy can be overcome by concurrent PD-L1 blockade.

Since these initial reports of clinical activity of PD-L1 blockade therapy in several tumor types, including advanced melanoma, lung, and renal cell carcinomas in 2012, there has been a growing interest in immunotherapy to see if these agents could be applied to other tumor subtypes. Furthermore, PD-L1 silencing with siRNA has been shown to inhibit colon cancer proliferation, migration, and invasion *in vitro* [48]. Increased PD-L1 expression in triple-negative breast cancer cells induced by PTEN shRNA loss led to decreased T-cell proliferation and increased apoptosis [36]. On the other hand, inhibition of PD-L1 also showed enhance NK and CD8(+) T cell-mediated immune function [49]. In conclusion, the present study shows that PD-L1 is highly expressed in chordoma. Expression of PD-L1 is also correlated with the prevalence of TILs in chordoma. This study suggests the potential of using PD-L1 based immunotherapy for the treatment of chordoma.

## MATERIALS AND METHODS

### Cell lines and cell culture

UCH1 and UCH2 are established human chordoma cell lines and were kindly provided by Dr. Silke Bruderlein (University Hospitals of Ulm, Germany) [34, 50]. Another human chordoma cell line, CH22, was established in our laboratory as previous reported [25]. These cell lines were cultured in the DMEM (Invitrogen, Carlsbad, CA) medium, supplemented with 10% fetal bovine serum (FBS) and 1% penicillin/streptomycin (Invitrogen). Cells were cultured in a humidified incubator with a 5% CO^2^-95% air atmosphere at 37°C and passaged every 3 - 4 days using trypsin-EDTA when cell confluence reached 90–100%.

Cells were treated with recombinant human interferon IFN-γ (Pierce Biotechnology, Rockford, IL), as previously described [51]. Briefly, 3 × 10^5^ cells were incubated at 37°C for 48 hours in medium supplemented with 100 U/mL, and then washed with excess culture medium.

### Western blot assay

Expression of PD-L1 protein was evaluated by Western Blot analysis. Protein lysates from chordoma cell lines were extracted using 1× Cell Lysis Buffer (Cell Signaling Technology, MA). The protein concentrations were determined using Protein Assay Reagents (Bio-Rad, CA) and a SPECTRAmax Microplate Spectrophotometer from Molecular Devices (Sunnyvale, CA). The primary antibodies for PD-L1 (1:1000 dilution), brachyury (1:1000 dilution), and β-actin (1:2,000 dilution) were purchased from Abcam, Santa Cruz, and Sigma-Aldrich, respectively. The secondary antibodies were bound to IRDye1 800CW or IRDye1 680LT (LI-COR Biosciences, NE). Western blots were carried out as previously described [52]. Normalization was performed using actin as an endogenous control. Membrane signals were scanned using an Odyssey infrared imaging system and analyzed using Odyssey 3.0 software (LI-COR Biosciences, NE). The protein levels were quantified with NIH Image J software.

### Human chordoma tumor tissues

Nine of the chordoma tissue samples (Tissue1–Tissue9) were obtained from the Massachusetts General Hospital Sarcoma Tissue Bank and were used in accordance with the policies of the institutional review board of the hospital (IRB protocol # 2007P-002464). Written informed consent was obtained from all patients whose specimens and clinical information were used for this research study. All tissue diagnoses were confirmed histologically, and proteins were extracted from these frozen tissues. The data of each patient's age, gender, metastasis, recurrence, tumor location(s), follow up months, and disease status were collected (Table 1). The expression of PD-L1 in these 9 chordoma samples was determined by Western blot assay.

**Table 1:** The clinical parameters of chordoma tissue microarray

parameters		n
Age		56
	<45	7
	45-60	16
	>60	30
	N/A	3
Gender		56
	Male	39
	Female	14
	N/A	3
Location		56
	Mobilespine	21
	Sacum	35
Sample status		78
	Primary	29
	Metastasis	8
	Local relapse	41
Disease status		56
	NED	24
	AWD	3
	DOD	22
	DOO	7

N/A:not applicable, NED: no evidence of disease, AWD: alive with disease, DOD: dead of disease, DOO: dead of others disease.

### Chordoma Tissue Microarray (TMA) and immunohistochemistry

Pathologically confirmed chordoma tissues were obtained from patients who had undergone surgical resection in our hospital, and were conducted according to the policies of the institutional review board of the hospital (IRB protocol # 2007P-002464). A retrospective study of 78 samples (including recurrent and metastatic) from 56 chordoma patients was identified for the tissue microarray (TMA) immunohistochemical staining as previously reported [53]. The clinical data of each patient's age, gender, tumor location(s), and disease status of chordoma patients were presented in the Table 2 (supplement data).

**Table 2:** Clinical data of protein samples from chordoma tissues

Sample	Age	Sex	Histologic Subtype	Metastatic	Recurrent	Location
1	48	M	Conventional	no	no	Sacrum
2	46	M	Conventional	no	no	Sacrum
3	74	M	Conventional	no	yes	Sacrum
4	60	F	Conventional	no	no	Sacrum
5	35	M	Conventional	yes	no	L1-L2
6	71	F	Conventional	no	no	Cervical
7	52	F	Conventional	no	no	Sacrum
8	74	M	Conventional	no	yes	Sacrum
9	52	M	Conventional	no	yes	Lumbar

The expression level of PD-L1 was determined based on the Immunohistochemistry Protocol (Paraffin) from Cell Signaling Technology (Beverly, MA). Briefly, 5-μm-thick array sections were baked at 60°C for 1h, dewaxed with xylene (three times for 5 minutes each), transferred through 100% ethanol (twice for 5 minutes each), rehydrated through graded alcohol, and then immersed in deionized water for 10 minutes. Antigen retrieval was processed with Target Retrieval Solution (Dako, North America, Inc., CA). After antigen retrieval, the slide was washed with PBS twice for 5 minutes. Following the process of antigen retrieval, endogenous peroxidase activity was quenched by incubation in 3% hydrogen peroxide. After protein blocking with blocking solution (Cell Signaling Technology) for 1 hour at room temperature, primary antibody (1:50 dilution, in 1% bovine serum albumin PBS) was applied at 4°C overnight in a humidified chamber. Primary PD-L1 antibody was probed at 4 °C overnight. Each step was succeeded by three Tris-buffered saline (TBS) rinses, and the bound antibody on the array was detected by using SignalStain® Boost Detection Reagent (Cell Signaling Technology) and SignalStain® DAB (Cell Signaling Technology). Finally, sections were counterstained with Hematoxylin QS (VectorLaboratories) and the slide was mounted with VectaMount AQ (Vector Laboratories) for long-term preservation. The immunostaining intensity pattern of PD-L1 was assessed on a scale semi-quantitatively as follows: 0, no staining; 1+, weak staining; 2+, moderate staining; and 3+, intense staining. Scoring was calculated from the mean of the two independently conducted assessments. PD-L1 staining images were obtained by using a Nikon Eclipse Ti-U fluorescence microscope (Nikon Corp) with a SPOT RT digital camera (Diagnostic Instruments Inc.).

### Evaluation of tumor-infiltrating lymphocytes

The TIL analysis were performed on the hematoxylin and eosin-stained chordoma TMA slide. Scores of the TMA were evaluated for the presence of TILs at 200× magnification and semi-quantitatively as follows: score 0, no TILs; 1, rare/few TILs; 2, brisk/prominent TILs.

### Statistical analyses

All statistical analyses were performed using GraphPad Prism software 5.0. Kaplan-Meier survival curves were generated to examine the relationship between the expression levels of PD-L1 and patients' survival rate. Survival time was calculated from the date of tumor diagnosis to the date of death or last follow-up. The statistical significance between two groups was determined using unpaired Student's t-test. Data were expressed as mean ± standard error of the mean (SEM). For comparison between expression of PD-L1 and immune infiltrates, a one-way ANOVA analysis was used. The statistical significance is described in figures and in legends. *P* values < 0.05 were considered as statistically significant.
